# GPR109A alleviate mastitis and enhances the blood milk barrier by activating AMPK/Nrf2 and autophagy

**DOI:** 10.7150/ijbs.62380

**Published:** 2021-10-17

**Authors:** Wenjin Guo, Wen Li, Yingchun Su, Shu Liu, Xingchi Kan, Xin Ran, Yu Cao, Shoupeng Fu, Juxiong Liu

**Affiliations:** College of Veterinary Medicine, Jilin University, Changchun 130062, China.

**Keywords:** Niacin, Mastitis, GPR109A, AMPK/Nrf2, Autophagy, Blood milk barrier

## Abstract

Mastitis causes great psychological and physical pain among women. Our previous studies found that niacin has anti-inflammatory effect, and the realization of this function depends on GPR109A. However, there are no previous reports about the anti-inflammatory function of GPR109A in mastitis. In our study, we observed the effect of niacin on the WT and GPR109A^-/-^ mice mastitis model. The results showed that administration of niacin to WT mice reduced the damage, proinflammatory mediators and protected the integrity of the blood milk barrier in mammary gland. While in GPR109A^-/-^ mice, there was no effect on the above indexes. In mammary epithelial cells, GPR109A was able to promote autophagy and Nrf2 nuclear import through AMPK. In LPS-induced mammary epithelial cells, niacin inhibited the LPS-induced inflammatory response and downregulation of tight junction proteins, and these effects were eliminated by knocking down GPR109A, blocking autophagy or inhibiting Nrf2 nuclear import. These results indicate that in mastitis, GPR109A promotes autophagy and Nrf2 nuclear import through AMPK, thereby inhibiting inflammatory damage to the mammary gland and repairing the blood milk barrier. Our results suggested that GPR109A may be a potential target for the treatment of mastitis.

## Introduction

Mastitis often occurs during lactation in women 1. Especially for the women who were give birth to a child for the first time, the incidence rate of mastitis in lactation was as high as 2-4% 2. When mastitis occurs in the breast, it causes local redness, swelling, pain and other symptoms 3. If not treated in time, mastitis can cause suppuration and septicemia, which endanger the life of affected women 4. Mastitis causes great physical and psychological pain, and the more advanced the disease, the stronger the pain 5. Therefore, timely prevention and control of mastitis can effectively reduce pain in women.

At present, the problem of how to most effectively treat mastitis in women in underdeveloped areas has not been solved, and mastitis still seriously affects the health of women in underdeveloped areas. Some studies have shown that mastitis can increase the risk of HIV transmission, especially in underdeveloped areas 6. Some studies have shown that women's reproductive health was affected by mastitis, so understanding mastitis and developing new treatments are of great importance.

The blood milk barrier formed by breast during lactation is an important barrier for the body 7, and it is closely related to the health of lactating women 8. Tight junction is an important part of the formation of blood milk barrier 9. Mastitis can severely damage the blood milk barrier 10. Wang Jingjing showed that mastitis caused by LPS can also seriously damage the blood milk barrier and aggravate inflammation 11. These studies have shown that breaking the blood milk barrier will lead to the aggravation of mastitis, and the aggravation of mastitis will also affect the blood milk barrier in turn. Therefore, how to protect the blood milk barrier and reduce the inflammatory reaction in the mammary gland is of positive significance to alleviate mastitis.

Niacin belongs to B vitamins and exists in animals and plants. And niacin is one of the 13 essential vitamins for the human body. Some studies reported that niacin can alleviate inflammatory response. Some studies have shown that niacin can reduce pulmonary hypertension through H-PGDs in macrophages, and some studies have shown that niacin can protect blood vessels by activating HO-1 *in vivo* and *in vitro* 12,13. These studies suggested that niacin may play an anti-inflammatory role in different parts of the body.

GPR109A is a G-protein-coupled receptor that regulates cellular activities by sensing various extracellular signals 14. Some studies have shown that GPR109A can alleviate TNBS-induced colitis by inhibiting p65 nuclear import 15. Other studies have shown that GPR109A can reduce the inflammatory response of microglia, thereby protecting neurons and alleviating Parkinson's symptoms in mice 16. These studies indicate that GPR109A has a good anti-inflammatory function and is expressed in many kinds of cells and tissues. In addition, GPR109A is highly expressed in the mammary gland 17. However, the anti-inflammatory function of GPR109A in the mammary gland has not been reported previously.

Nrf2 plays an important role in redox reaction 18,19. Nrf2 can reduce cell damage caused by oxidative stress and maintain cell homeostasis 20,21. Previous studies have shown that insufficient niacin intake significantly reduces Nrf2 expression and increases the expression of proinflammatory mediators in young grass carp 22. However, there are no available reports on the activation of Nrf2 by niacin in mammals.

Autophagy is an important means for cells to maintain normal life activities, and autophagy is involved in various inflammatory reactions and barrier repair 23,24. MiR-155 can alleviate lung injury by regulating autophagy 25. It has also been shown that induction of autophagy can alter tightly linked scaffold proteins 26. So autophagy is closely related to inflammation and the blood milk barrier, but there are few studies on the role of autophagy in mastitis or its role in maintaining the blood milk barrier. Therefore, we speculate that GPR109A may alleviate inflammation and enhance the blood milk barrier by activating autophagy and promoting Nrf2 nuclear import.

## Materials and methods

### Reagents

3-methyladenine (3-MA) and Dorsomorphin (Compound C, CC) were supported from Selleckchem (Shanghai, China). ML385 was purchased from MedChemExpress (USA). Niacin was purchased from sigma (USA).

### Animal experiments

In our experiments, All GPR109A^-/-^ and wild-type (WT) pregnant female mice (C57BL/6) were 9-week-old, The GPR109A^-/-^ mice is a generous gift from Dr. Martin Sager in Germany. The experiments were approved by the Jilin University Institutional Animal Care and Use Committee.

The whole animal experiment was divided into 8 groups. WT mice included NT group, niacin group, LPS group and niacin + LPS group, and GPR109A^-/-^ mice also included the above four groups. Then prepare purified water containing 50 mM niacin to ensure the freedom of diet and drinking water for mice. At the beginning of the experiment, the niacin group and niacin + LPS group in WT and GPR109A^-/-^ mice were fed with purified water containing niacin until the end of the experiment. WT mice and GPR109A^-/-^ were caged respectively. One male mouse and two female mice were caged for one week. After pregnancy, the female rats were divided into groups and fed purified water containing niacin. The pregnancy cycle of mice was 21 days, and the experiment was carried out 6 days after birth. Therefore, the whole niacin feeding cycle was 27 days. On day 26, the mice were anesthetized and LPS was injected into the fourth pair of inguinal mammary glands of the mice. The mice were then put back into the cage and the mammary glands were collected 24 hours later. During this period, they are free to eat and drink water, and the drinking water is purified water containing niacin. The suckling mice were fed to other female mice.

### Protein levels of MDA, TNF-α, IL-6 and IL-1β

Firstly, the mouse mammary gland tissue was ground and the supernatant was collected, and then the contents of TNF-α, IL-6 and IL-1β in the supernatant were detected. All detection steps refer to the instructions in the ELISA kit (CA, Biolegend, Cat: 430904, Cat: 431304, Cat: 432604), and the addition amount of supernatant is adjusted according to the actual situation.

### Myeloperoxidase (MPO) activity assay

The collected fresh mouse mammary glands were ground and the supernatant was collected. MPO content in mouse mammary gland was detected through the previous test steps of our research group 27.

### Evaluation of histological changes

H&E staining procedure and pathological score in this study were according to previously described 28.

### Construction of GPR109A knockdown plasmid

The construction of GPR109A knockdown plasmid (GPR109A-shRNA) was carried out by Songon Biotech (Shanghai, China). Four sequences of GPR109A knockdown plasmids were designed. We found that the knockdown effect of shRNA1 was the best among these plasmids through pre-experiment (S 2).

### Cell culture

EpH4-Ev cells were cultured in DMEM medium (Gibco, Grand Island, NY, USA) containing 10% fetal bovine serum (Hyclone, USA) at 37 °C in a humidified incubator with 5% CO_2_.

### The effect of niacin on Eph4-Ev activity was detected by Cell counting kit-8 assay

Eph4-Ev cells were inoculated into 96 well plates and added 100 μL culture medium. After stimulation with LPS or niacin for 24 hours, CCK-8 reagent was added to the well which inoculated with cells, and the proportion of addition refers to the instructions. After waiting for 1 hour, detect the absorbance with 450 nm wavelength.

### Detection of barrier permeability by FITC-albumin

To verify the integrity of the blood milk barrier, fresh mammary gland tissue was immersed in PBS containing FITC-albumin. After 20 minutes, the mammary gland tissue was immersed in liquid nitrogen, and the frozen section was performed. Then the green fluorescence in the section was observed under fluorescence microscope. The detailed steps refer to the previous protocol 11.

### The mRNA level in mammary gland tissue or mammary epithelial cells was detected by qRT-PCR

We added TRIzol to collected mammary tissue or Eph4-EVs. Then, the total RNA in mammary tissue or Eph4-EVs was collected by a series of treatments such as chloroform, isopropanol and alcohol. Finally, the collected RNA was reverse transcribed into cDNA. The experimental method of reverse transcription was descripted previously 27. Three independent operations were performed in each experiment. The primers we designed are shown in Table 1.

### Immunofluorescence

We quickly frozen the fresh mammary tissue in liquid nitrogen, then treated the tissue block with OCT, and put it into a frozen slicer for slicing after OCT solidification. The frozen sections were stained with immunofluorescence according to the previous steps 27. The primary antibodies used in this study were diluted as follows: anti-Nrf2 (1:150), anti-Claudin 3 (1:200), anti-ZO-1 (1:200) and anti-Occludin (1:200). All immunofluorescence staining will be observed and photographed by fluorescence microscope.

### Electron microscopy

The operation procedure and method of TEM were all referred to the previous description 29.

### Plasmids and fluorescence microscopy

Plasmid of mRFP-GFP-LC3 was transfected into the EpH4-Evs by LipoFiter (HANBIO), and then observed by laser confocal microscope.

### Co-immunoprecipitation

We first collected the total protein of Eph4-ev cells, and then operated according to the instructions of Pierce^TM^ Classic Magnetic IP/Co-IP Kit (Thermo Fisher, USA).

### The protein was detected by Western blotting

Firstly, mammary tissue or Eph4-EVs were lysed with protein lysate to collect total protein. Then Pierce BCA Protein Assay Kit (Thermo Fisher, USA) was used to detect the concentration of the extracted total protein, add appropriate 5×SDS according to the protein concentration, and finally boil for 5 minutes to denature the protein. Western blotting was performed using standard protocols. The primary antibodies used are shown in Table 2.

### Statistical analysis

All images and analyses were performed using GraphPad Prism software (La Jolla, CA, USA). All data were expressed as mean ± SD. When the grouping is greater than or equal to 3, we use Oneway-ANOVA method for comparison. When the overall F test was significant (*P* < 0.05), Tukey's adjustment method was used for post comparison to determine the degree of significant pairwise difference.

## Results

### Binding site of niacin with GPR109A

In order to study the biological function of niacin *in vivo* (S1d). We simulated the binding site between niacin and GPR109A, and found that niacin can activate GPR109A receptor through SER-244 and THR-280, thus playing its biological function (S1a-c).

### GPR109A alleviate pathological injury to the mammary gland

**Figure 1:**
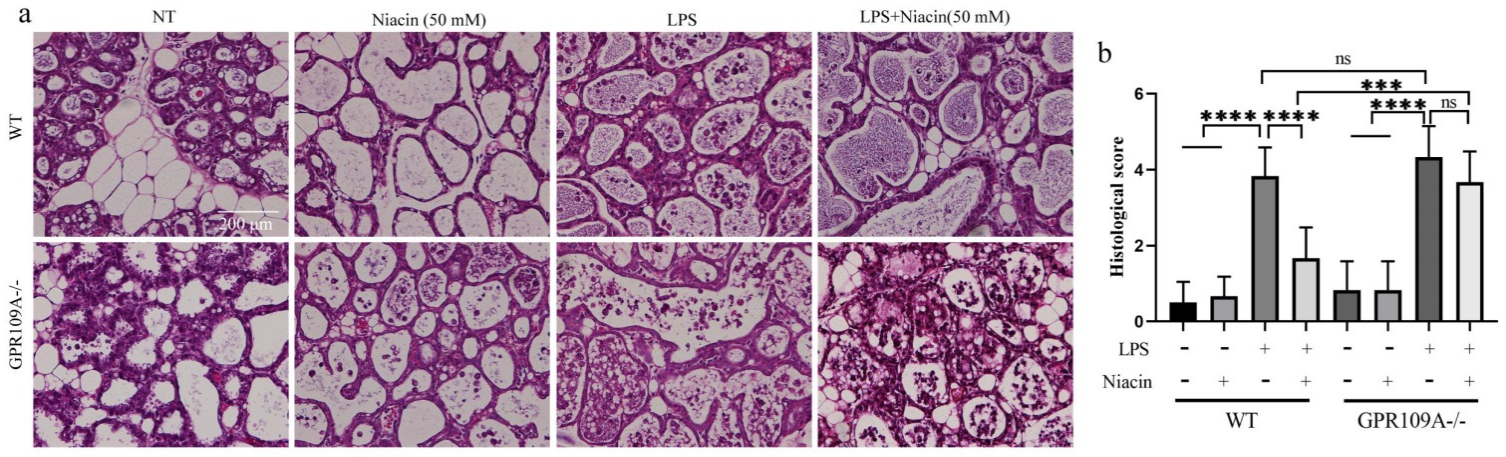
** Effects of GPR109A on mice mammary gland injury. (a)** The H&E staining of mammary gland (original magnification 200 ×). **(b)** The injury score of mammary gland, this is based on the three points previously described.Values are presented as means ± SD (*** *p*<0.001, **** *p*<0.0001).

We performed sections and H&E staining of mammary tissue (Fig. 1a). In WT and GPR109A^-/-^ mice, there was no damage to the mammary glands in the NT and Niacin groups (Fig. 1a). Compared with those from the NT and Niacin group, mammary glands from the LPS group presented with severe pathological injuries, such as thickening of the alveolar wall and neutrophil infiltration (Fig. 1a). Neutrophil infiltration in the LPS group of GPR109A^-/-^ mice was more serious than that in the LPS group of WT mice. Neutrophil infiltration in the LPS + niacin group in WT mice was significantly improved after niacin treatment, but the same effect was not observed in LPS + niacin group in GPR109A^-/-^ mice (Fig. 1a). The pathological scores of these mammary glands were also consistent with the results of H&E staining (Fig. 1b).

### GPR109A decreased the expression of proinflammatory cytokines

**Figure 2:**
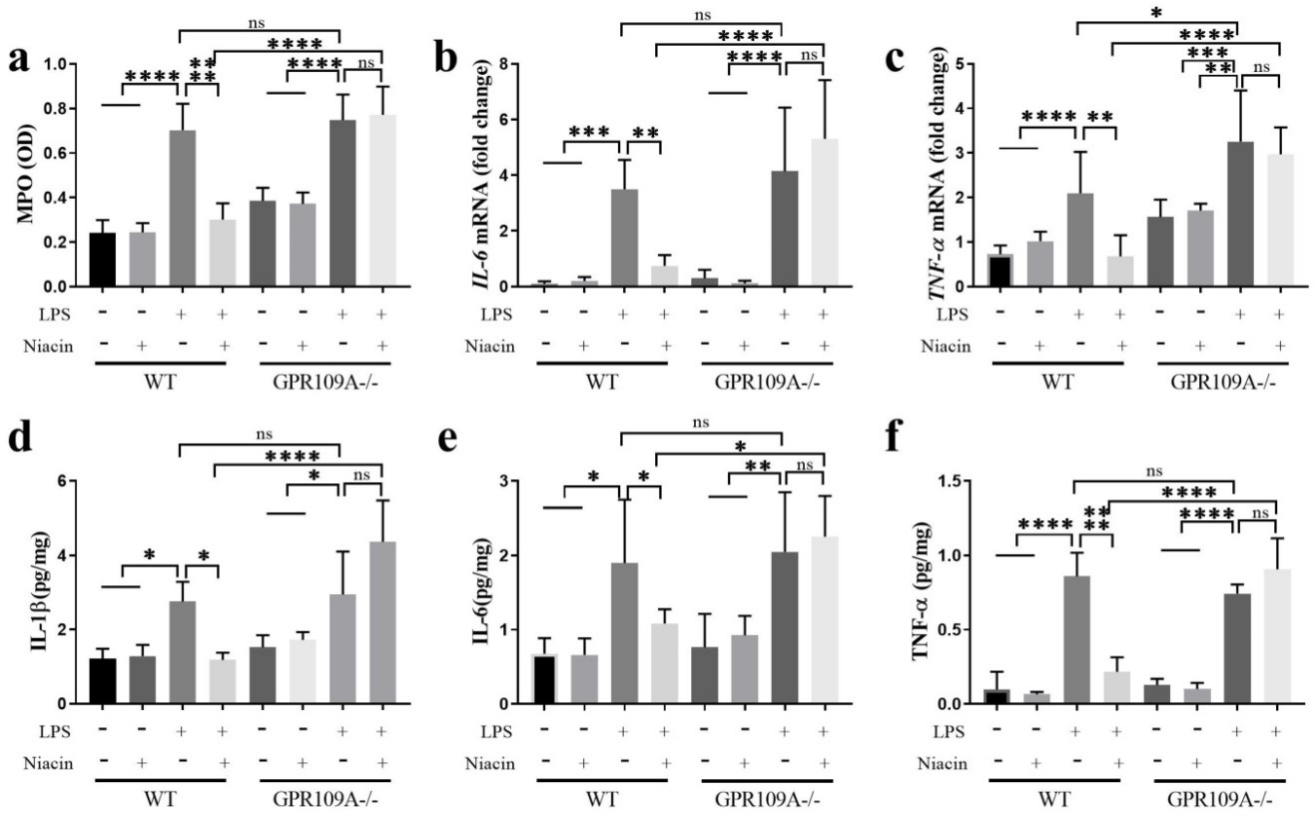
** Activating GPR109A can reduce the release of cytokines. (a)** Activity of myeloperoxidase (MPO). **(b, c)** mRNA levels of *IL-6* and* TNF-α*. **(d-f)** The Protein contents of IL-1β, TNF-α and IL-6. Values are presented as means ± SD (n = 5) (* *p*<0.05, ** *p*<0.01, *** *p*<0.001, **** *p*<0.0001).

Mammary epithelial cells are opportunistic sentinel cells that play an important role in monitoring the invasion of the mammary gland by pathogenic microorganisms 30. In WT mice, the levels of MPO, IL-1β, IL-6, and TNF-α in mammary gland tissues were higher than other groups (Fig. 2a-f). Compared with the LPS group, niacin treatment remarkably reduced the expression levels of these inflammatory factors (Fig. 2a-h). In GPR109A^-/-^ mice, the MPO, IL-6, IL-1β and TNF-α levels were increased in the LPS and LPS+niacin groups (Fig. 2a-f). The level of *TNF-α* in GPR109A^-/-^ mice was significantly higher than that in WT mice (Fig. 2c).

### GPR109A repairs the integrity of the blood-milk barrier

**Figure 3:**
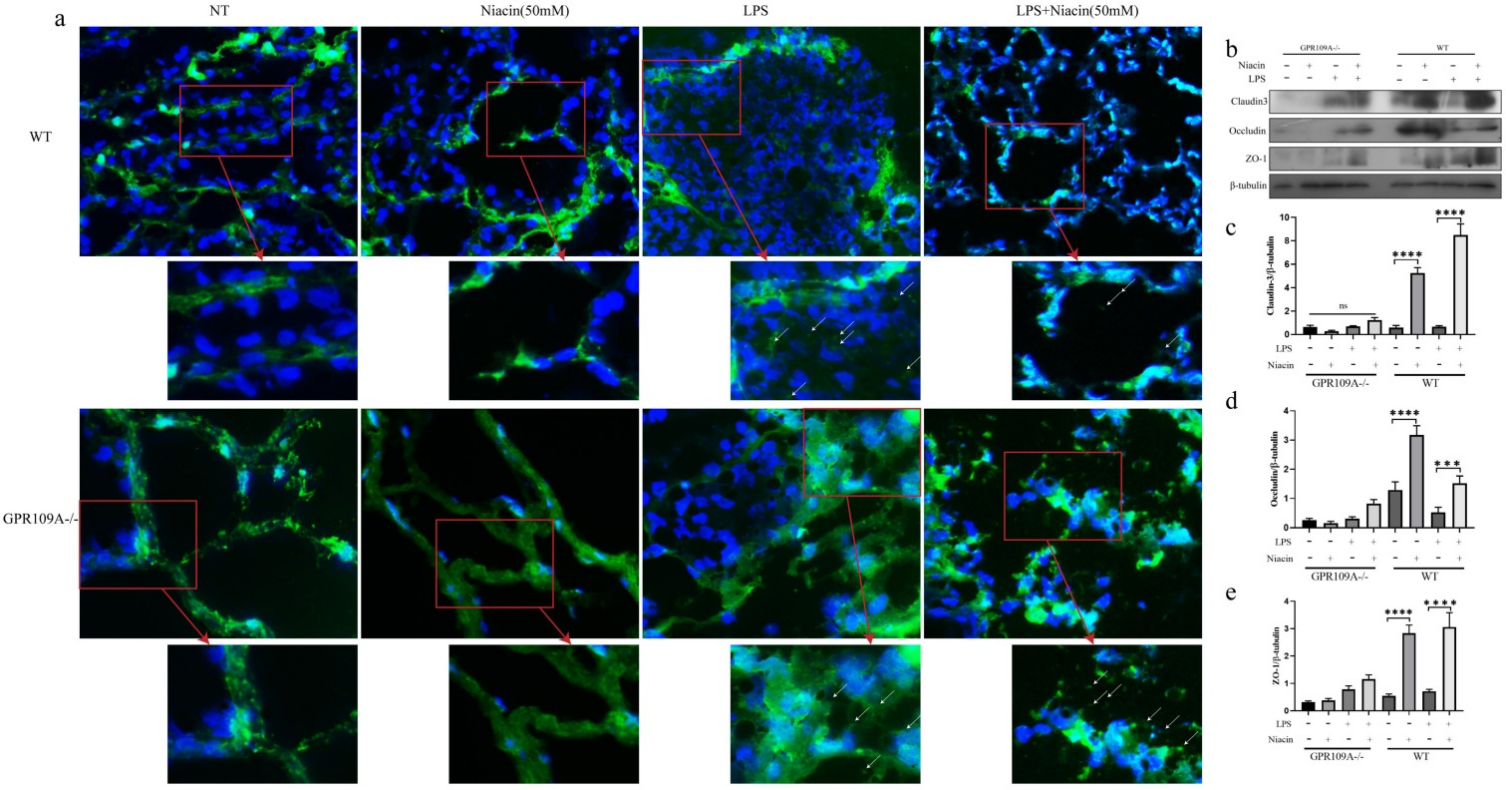
** Penetration of FITC-albumin as it enters mammary acini through the blood milk barrier.** Mammary glands from the eight groups were treated with previous description. **(a)** The color green represent FITC-albumin and the colot blue means nuclei (DAPI). After LPS injection, we can observe green FITC-albumin in mammary acini under fluorescence microscope. **(b-e)** The levels of Occludin, Claudin 3 and ZO-1. Values are presented as means ± SD (*** *p*<0.001, **** *p*<0.0001).

Blood milk barrier is very important in lactating women 31. In WT mice, FITC-albumin penetrated into the acini in LPS group (Fig. 3a). As expected, niacin could inhibit the LPS-induced barrier damage (Fig. 3a).

In GPR109A^-/-^ mice, there was no fluorescence in acinar lumina in the NT group or Niacin group, but there was a large amount of FITC-albumin in acinar lumina in the LPS group, more than that present in WT mice. After the niacin treatment, we found that there was still a large amount of FITC-albumin in the acinar lumina of GPR109A^-/-^ mice (Fig. 3a).

To further study the effect of GPR109A on the blood milk barrier, we detected the levels of ZO-1, Occludin and Claudin 3. In WT mice, the levels of ZO-1, Occludin, and Claudin 3 in the LPS group were significantly downregulated, while the levels of these proteins was significantly increased after niacin treatment. In GPR109A^-/-^ mice, we found that the expression of Claudin 3, ZO-1 and Occludin was very low (Fig. 3b-e). These results suggested that GPR109A may be involved in maintaining the blood milk barrier.

**Figure 4:**
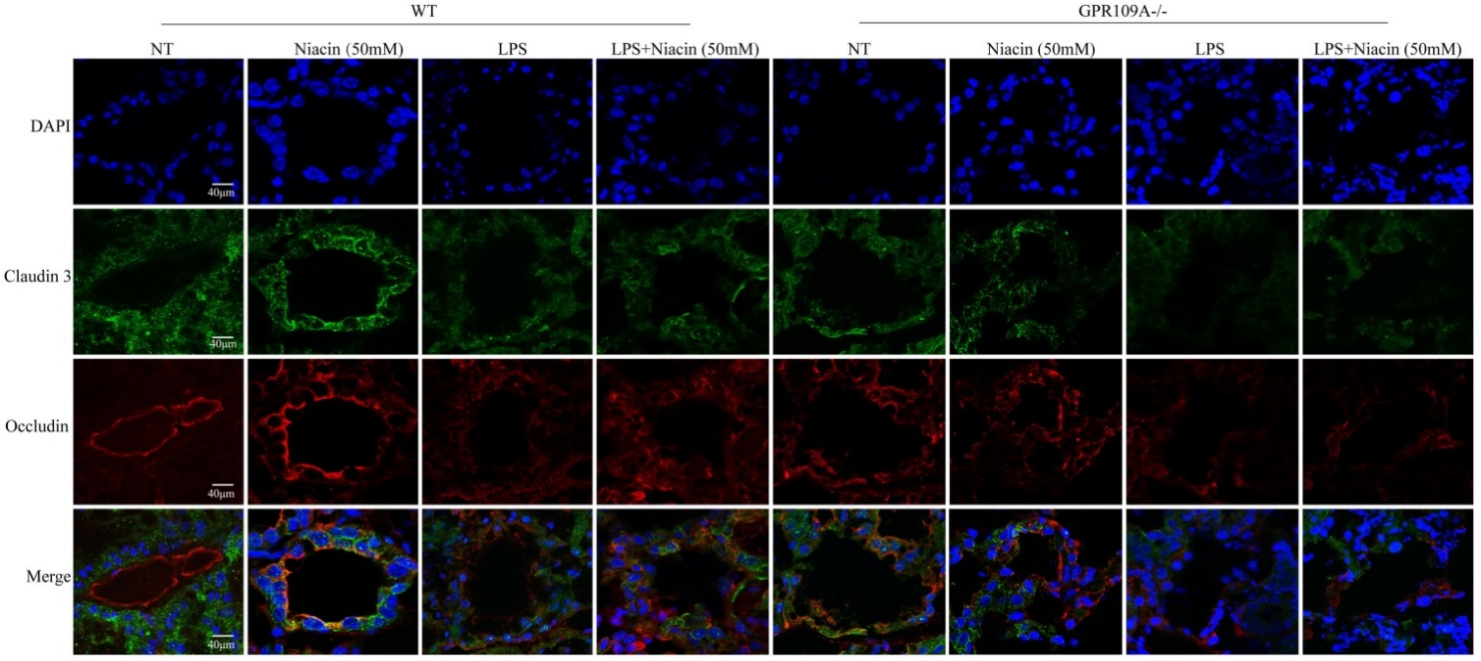
** Immunofluorescence results of Claudin 3 and Occludin.** Immunofluorescence staining was performed on 8 groups of mammary glands, and the colocalization and fluorescence intensity of Claudin 3 and Occludin in mammary glands were determined.

**Figure 5:**
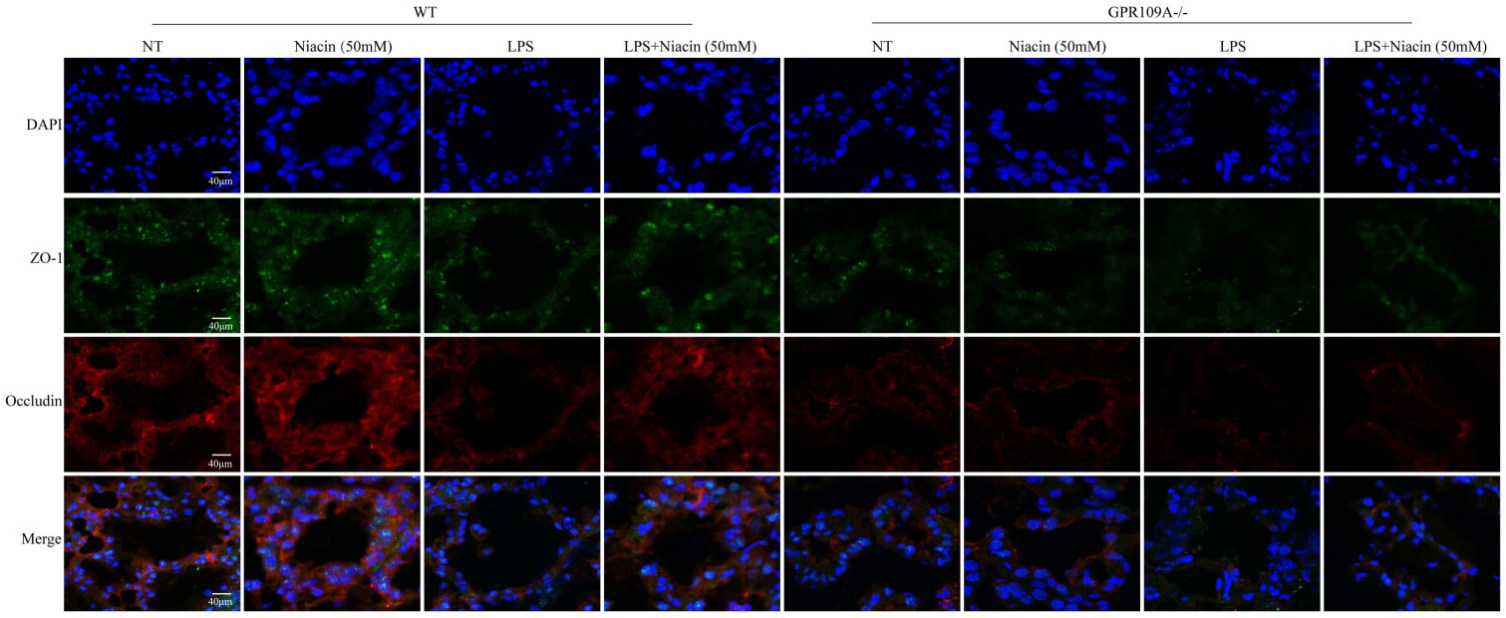
** Immunofluorescence results of ZO-1 and Occludin.** Immunofluorescence staining was performed on 8 groups of mammary glands, and the colocalization and fluorescence intensity of ZO-1 and Occludin in mammary glands were determined.

Tight junction is an important structure of blood-milk barrier, and they can limit the invasion of microorganisms and regulate the exchange of various substances in the mammary gland. To clarify the mechanism of how GPR109A helps repair the integrity of the blood-milk barrier. In WT mice, the fluorescence intensity of Claudin-3, ZO-1 and Occludin in LPS + niacin group was significantly stronger than that in LPS group, suggesting that niacin repairs tight junction destroyed by LPS (Fig. 4 & Fig. 5). At the same time, we also found that LPS significantly reduced the fluorescence intensity of ZO-1, occludin and Claudin-3 in mammary gland. Before LPS injection, Claudin-3 and ZO-1 were co-located with occludin in the most-apical region of mammary epithelial cells (MEC) (Fig. 4 & Fig. 5). We also found that ZO-1 and Claudin-3 in the most-apical region of the junction of MECs could not co-locate with Occludin after injection of LPS 24 hours (Fig. 4 & Fig. 5). After feeding nacin, the blood milk barrier of LPS injected WT mice was significantly repaired, the expression of ZO-1, Claudin-3 and Occludin was significantly increased, and their localization was partially restored. In GPR109A^-/-^ mice, the fluorescence intensities of ZO-1, Claudin-3 and Occludin were weaker than those in WT mice (Fig. 4 & Fig. 5). Moreover, we found that the location of ZO-1, Claudin 3 and Occludin in GPR109A^-/-^ mice's mammary gland were changed (Fig. 4 & Fig. 5). After LPS injection, niacin could not restore the expression of above proteins in GPR109A^-/-^ mice.

### GPR109A enhances autophagy of mammary epithelial cells

**Figure 6:**
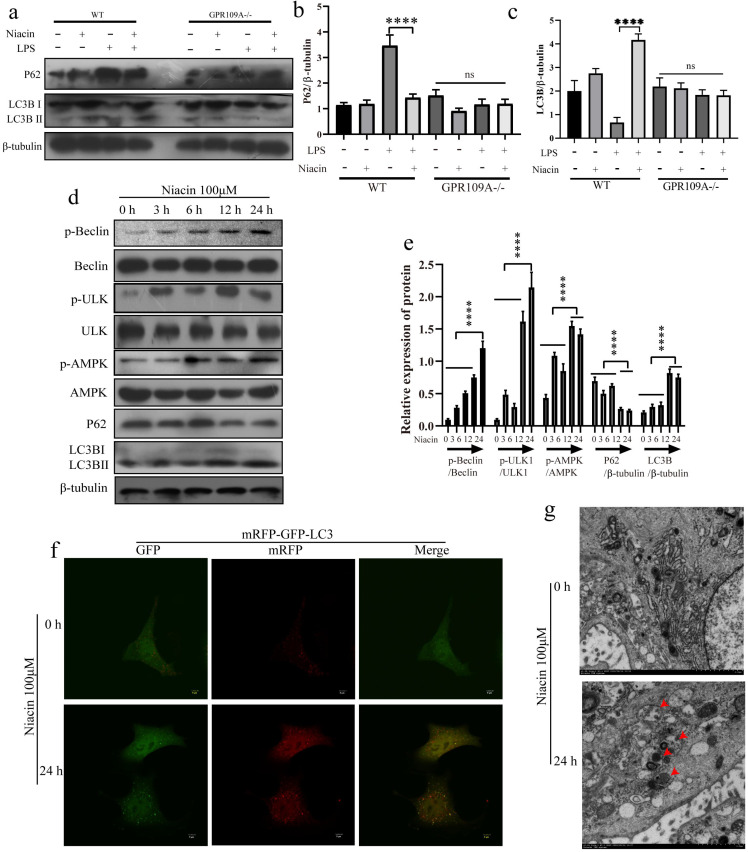
** Effect of GPR109A on autophagy. (a-c)** Protein levels of p62 and LC3B in the mammary gland. **(d, e)** Protein levels of p-APMK, AMPK, p-Beclin, Beclin, p-ULK1, ULK1, P62 and LC3B in niacin treated cells at 0, 3, 6, 12 and 24 hours. **(f)** After 24 hours of niacin treatment, the autophagy flow of EpH4-Ev increased significantly. **(g)** Autophagy lysosomes in EpH4-Ev. Values are presented as means ± SD (n = 3) (**** *p*<0.0001).

*In vivo*, we also found that the protein levels of P62 and LC3B in the mammary glands of GPR109A^-/-^ mice significantly decreased, while the protein levels of P62 and LC3B significantly increased after GPR109A was activated by niacin in WT mice (Fig. 6a-c). To further study the function of GPR109A, we treated EpH4-Ev cells with niacin for 0, 3, 6, 12 or 24 h. Our results showed that niacin could activate autophagy. Niacin enhances autophagy, degradation of P62 and expression of LC3B by promoting the phosphorylation of AMPK, Beclin and ULK1 (Fig. 6d, e). When the mRFP-GFP-lc3 plasmid was transfected into cells, the flow of autophagy was significantly enhanced after treatment with niacin for 24 h (Fig. 6f). The electron microscopy results also showed that niacin could significantly promote the formation of autophagic lysosomes (Fig. 6g).

### GPR109A activates autophagy through nonclassical pathways

**Figure 7:**
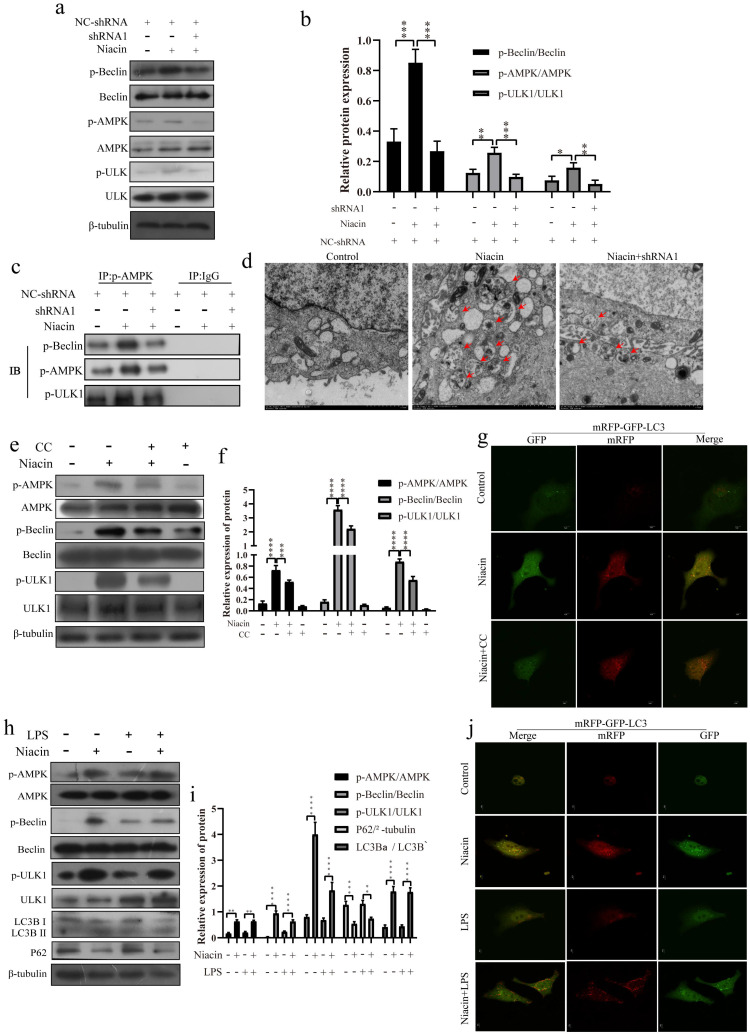
** GPR109A inhibits inflammation by activating autophagy through p-AMPK, p-Beclin and p-ULK1. (a, b)** The levels of p-AMPK, p-ULK1 and p-Beclin after knocking down GPR109A. **(c, d)** Interaction between p-ULK1, p-Beclin and p-AMPK after knocking down GPR109A. **(e-g)** Protein levels of p-ULK1 and p-Beclin after inhibition of AMPK. **(h)** Protein levels of the autophagy related pathway proteins after GPR109A was activated in LPS-induced MECs. **(j)** Autophagy flow in the LPS-induced MECs after GPR109A activation. Values are presented as means ± SD (n = 3) (*p<0.05, **p<0.01, ***p<0.001, ****p<0.0001).

Studies have shown that autophagy can alleviate inflammation and enhance TJs 32,33. To further study the anti-inflammatory mechanism of GPR109A, we detected the phosphorylation levels of AMPK, Beclin and ULK1 after knocking down GPR109A. Our results showed that activation of GPR109A promoted phosphorylation of AMPK, Beclin and ULK1 (Fig. 7a, b). The IP test results showed that p-AMPK may be the main factor affecting the phosphorylation of Beclin and ULK1 and that the interaction of p-AMPK, p-Beclin and p-ULK1 is also closely related to the activation of GPR109A (Fig. 7c). The results of the IP test were consistent with those of electron microscopy. Activation of GPR109A significantly promoted the formation of autophagic lysosomes (Fig. 7d). To further prove that AMPK is the main downstream effector protein of GPR109A, we inhibited it. After inhibition of AMPK, phosphorylation of AMPK, Beclin and ULK1 was also significantly inhibited (Fig. 7e, f). The autophagy flow results were consistent with the western blotting results (Fig. 7g). These results indicate that GPR109A mainly promotes autophagy through AMPK.

To further study the mechanism of GPR109A in alleviating inflammation, we constructed an LPS induced inflammatory response model of EpH4-Ev. After activating GPR109A, the mechanism of GPR109A in the inflammatory response model was studied. We found that activation of GPR109A significantly promoted the phosphorylation of autophagy related proteins (AMPK, Beclin and ULK1) and the degradation of p62 and transformation of LC3BⅠ into LC3BⅡ (Fig. 7h, i). After transfection of the mRFP-GFP-LC3 plasmid, we found that GPR109A activation significantly enhanced autophagy (Fig. 7j).

### GPR109A can activate the Nrf2 signaling pathway

**Figure 8:**
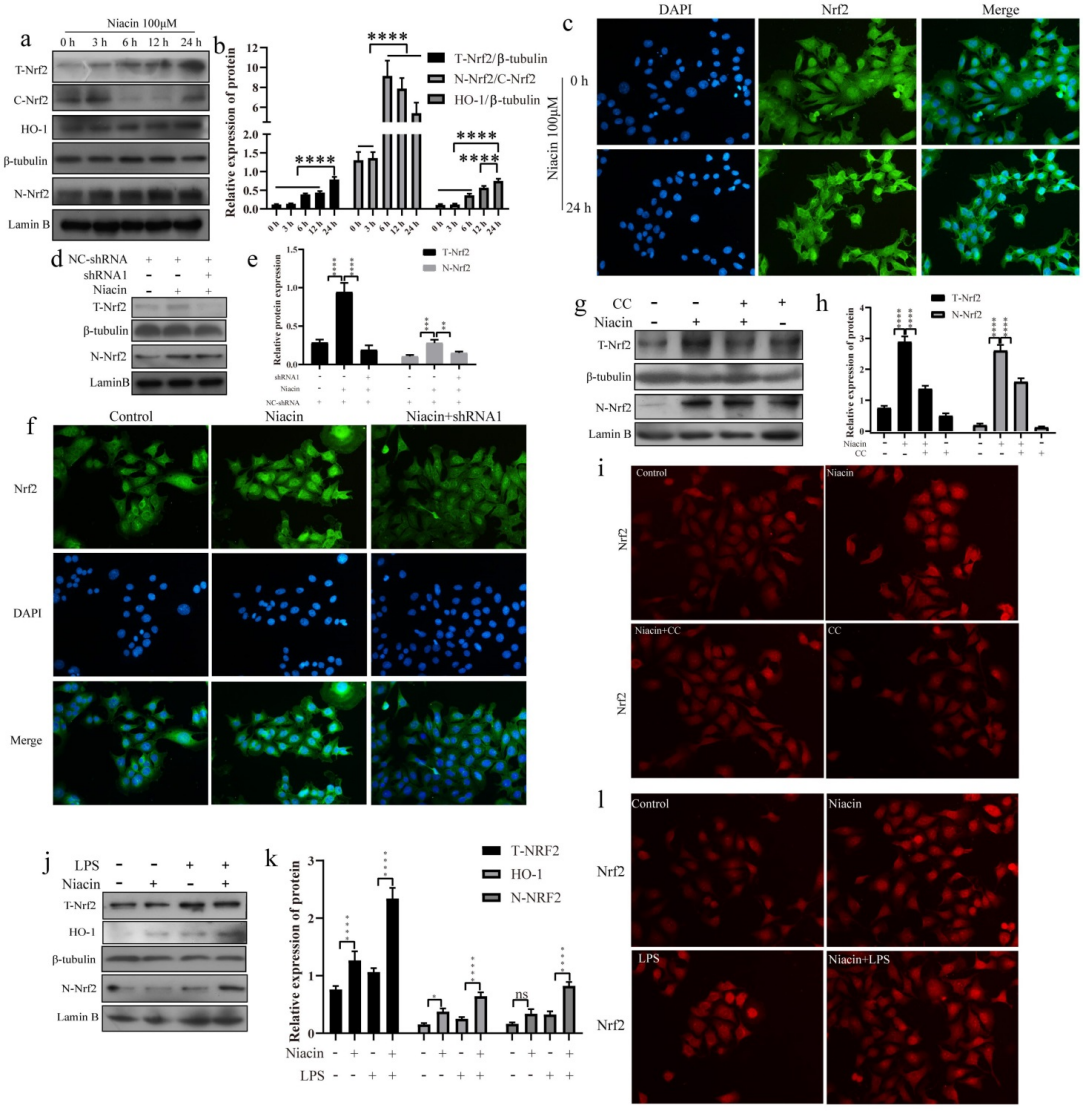
** GPR109A promotes Nrf2 nuclear import to exert anti-inflammatory effect by phosphorylating AMPK. (a, b)** Protein levels of T-Nrf2, C-Nrf2, HO-1 and N-Nrf2. **(c)** Immunofluorescence results of Nrf2 nuclear import after treating with niacin for 24 hours. **(d, e)** The levels of N-Nrf2 and T-Nrf2. **(f)** Immunofluorescence results of Nrf2 nuclear import after knocking down GPR109A. **(g, h)** The levels of N-Nrf2 and T-Nrf2. **(i)** Immunofluorescence results of Nrf2 nuclear import after treating with niacin, CC or niacin+CC for 24 hours. **(j, k)** Protein levels of T-Nrf2, HO-1 and N-Nrf2. **(l)** Immunofluorescence results of Nrf2 nuclear import after treating with LPS, niacin or niacin+LPS for 24 hours. Values are presented as means ± SD (n = 3) (*p<0.05, **p<0.01, ***p<0.001, ****p<0.0001).

We also found that GPR109A can promote Nrf2 nuclear import. To further study the function of GPR109A, we treated EpH4-Ev cells with niacin for 0, 3, 6, 12 or 24 h. Our results showed that niacin could promote the expression of T-Nrf2 (Total-Nrf2), N-Nrf2 (Nuclear-Nrf2) and HO-1 (Fig. 8a, b). After 24 h, the fluorescence intensity in the nucleus was significantly higher than that at 0 h (Fig. 8c). After GPR109A knockdown, the expression of T-Nrf2 and N-Nrf2 significantly decreased (Fig. 8d, e). The immunofluorescence results also showed that Nrf2 nuclear import was significantly inhibited after GPR109A was knocked down (Fig. 8f). Interestingly, T-Nrf2 and N-Nrf2 expression also significantly decreased after AMPK inhibition (Fig. 8g, h). The immunofluorescence results were consistent with the western blotting results (Fig. 8i), suggesting that GPR109A might regulate the nuclear import of Nrf2 through the AMPK signaling pathway. We also verified this hypothesis in the model of the inflammatory response of mammary epithelial cells constructed by treatment with LPS. The immunofluorescence and western blot results showed that the LPS + niacin group had significantly enhanced Nrf2 nuclear import and increased expression of HO-1, thus playing an anti-inflammatory role (Fig. 8j-l).

### GPR109A plays an anti-inflammatory role through Nrf2 and autophagy

**Figure 9:**
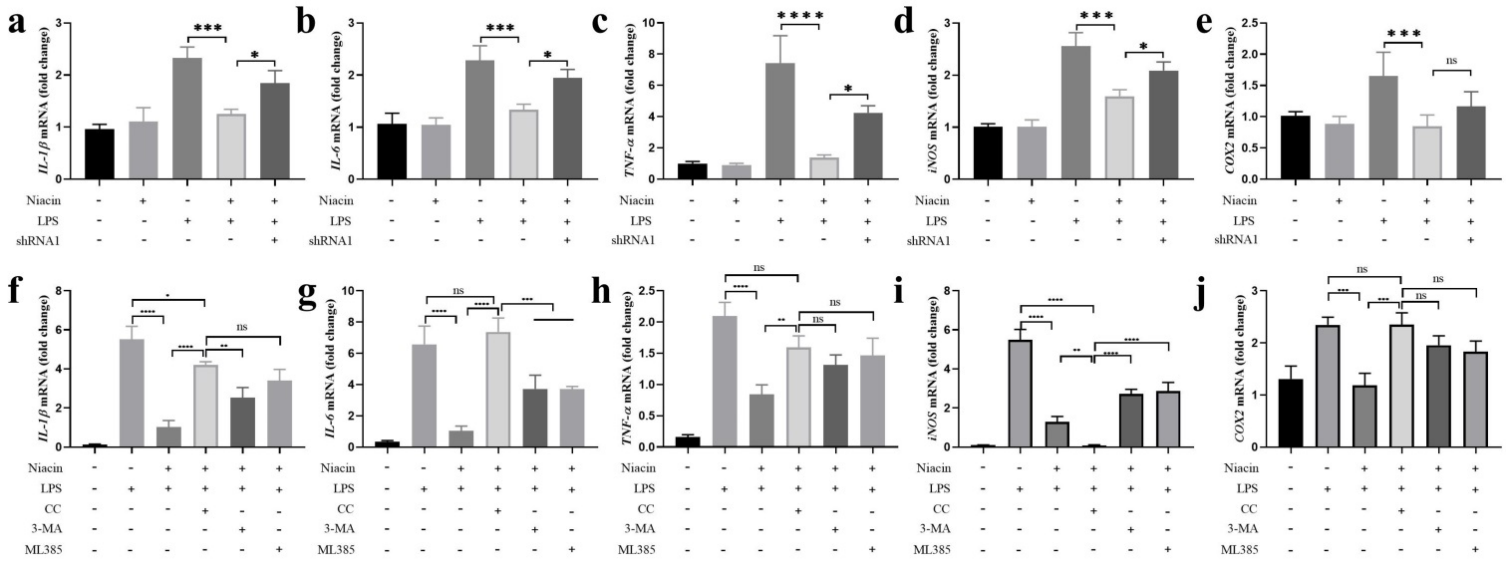
** GPR109A could exert anti-inflammatory effect by activating autophagy and Nrf2 nuclear import through AMPK signal pathway. (a-e)** The levels of *TNF-α, IL-1β, IL-6*,* COX-2* and *iNOS* were detected using qRT-PCR in EpH-Ev (n=3)*.* **(f-j)** Gene levels of *TNF-α, iNOS, COX-2, IL-6* and *IL-1β* were detected using qRT-PCR in EpH-Ev (n=3)*.* Values are presented as means ± SD (*p<0.05, **p<0.01, ***p<0.001, ****p<0.0001).

The release of proinflammatory mediators could aggravate inflammation 34. To further explore the important function of GPR109A in anti-inflammation, we detected proinflammatory mediators in the Eph4-Ev cell line. The results showed that niacin significantly reduced the mRNA expression levels of *TNF-α, IL-1β, IL-6*, *COX-2* and *iNOS* after 24 h of niacin treatment, and the anti-inflammatory effect of niacin was significantly weakened after GPR109A was knocked down. These results indicated that GPR109A plays an important anti-inflammatory role after activation (Fig. 9a-e). Moreover, we found that the expression of *IL-1β, IL-6, TNF-α* and *COX-2* was significantly increased after AMPK inhibition, but not *iNOS* (Fig. 9f-j). However, the expression of these inflammatory genes increased slightly when autophagy and Nrf2 were inhibited (Fig. 9f-j). These results suggest that GPR109A alleviates inflammation through the AMPK/Nrf2 and AMPK/autophagy signaling pathways.

### GPR109A enhances the tight junction (TJ) of mammary epithelial cells

**Figure 10:**
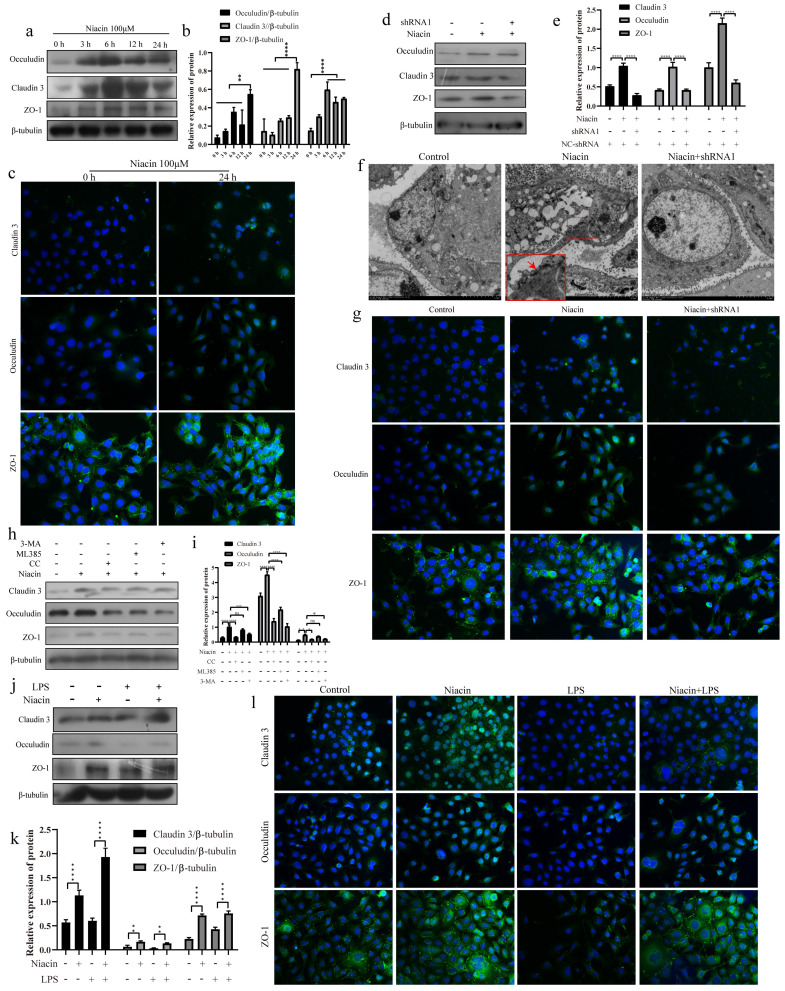
** GPR109A enhances tight junction protein expression by activating autophagy and Nrf2. (a, b)** The levels of ZO-1, Claudin 3 and Occludin after niacin treatment for 0, 3, 6, 12 and 24 hours. **(c)** Immunofluorescence results of Claudin 3, ZO-1 and Occludin after niacin treatment for 24 hours. **(d, e)** The levels of ZO-1, Claudin 3 and Occludin after knocking down GPR109A. **(f)** The results of electron microscopy show that the tight junction was strengthened after adding niacin. **(g)** Immunofluorescence results of Claudin 3, ZO-1 and Occludin after knocking down GPR109A. **(h, i)** The levels of Occludin, Claudin 3 and ZO-1 in EpH-Ev cells that were treated with niacin, niacin+3-MA, niacin+ML385 or niacin+CC. **(j, k)** The levels of Occludin, Claudin 3 and ZO-1 after the cells were treated with LPS, niacin or niacin+LPS. Values are presented as means ± SD (n=3) (*p<0.05, **p<0.01, ***p<0.001, ****p<0.0001).

To explore the effect of GPR109A on TJs, we detected the protein expression and fluorescence intensity of Occludin, ZO-1 and Claudin 3. The results showed that the expression of Occludin, Claudin 3 and ZO-1 were significantly increased after niacin treatment (Fig. 10a, b). The fluorescence intensities of Occludin, ZO-1 and Claudin-3 significantly increased after niacin treatment for 24 h (Fig. 10c). These results indicate that the levels of Occludin, Claudin-3 and ZO-1 in mammary epithelial cells significantly increased after GPR109A was activated and significantly decreased after GPR109A was knocked down (Fig. 10d, e). The fluorescence intensity of these proteins was consistent with the western blotting results (Fig. 10g). The transmission electron microscope (TEM) results also showed that the TJs were strengthened after GPR109A was activated (Fig. 10f).

To further understand how GPR109A enhances TJs, we added inhibitors of autophagy (3-MA, 5 µM), AMPK (CC, 5 µM) and Nrf2 (ML385, 5 µM). The results showed that inhibition of AMPK and Nrf2 significantly reduced the protein levels of the TJs, indicating that the AMPK signaling pathway and autophagy play important roles in TJs (Fig. 10h, i). Nrf2 could regulate the expression of Occludin (Fig. 10 h, i).

In MECs, we also found that GPR109A could protect the integrity of the blood milk barrier and promote the expression of TJs. The results showed that the levels of Claudin 3, ZO-1 and Occludin in the LPS + niacin group significantly increased (Fig. 10j, k). The immunofluorescence results also showed that GPR109A could promote the expression of TJs (Fig. 10l) and that this function was mainly mediated through the AMPK/autophagy signaling pathway.

## Discussion

In this study, we found that the integrity of the blood milk barrier and the level of autophagy of the GPR109A^-/-^ mice's mammary gland significantly decreased and that the inflammatory response of the GPR109A^-/-^ mice significantly increased after LPS stimulation. These results showed that GPR109A plays important roles in improving the blood milk barrier, in alleviating inflammation and in changing autophagy. Our other experiments showed that GPR109A could regulate the phosphorylation of ULK1, Beclin and AMPK to improve mastitis and enhance the blood milk barrier. Moreover, GPR109A could also promote the expression of Nrf2 and HO-1, thus playing an anti-inflammatory role. Therefore, the results of our study suggested that GPR109A might alleviate inflammation and enhance the blood milk barrier by activating autophagy and promoting Nrf2 nuclear import.

Autophagy is an important means by which cells cope with the internal and external environments 35. When cells sense changes in their internal and external environment, they can maintain homeostasis and the healthy operation of organelles by regulating their autophagy level 36. When mammary epithelial cells are stimulated by LPS, they produce a strong inflammatory response and release a large number of proinflammatory mediators 37. These proinflammatory mediators may change the microenvironment inside and outside cells 38, destroy the blood milk barrier, and intensify the inflammatory response 39. Some studies have shown that autophagy can maintain the healthy operation of cells 40, enhance the blood milk barrier and reduce the inflammatory response by clearing aging and damaged organelles 41,42. It has been shown that activation of GPR109A can reduce inflammation and improve the symptoms of ulcerative colitis 15. Although the anti-inflammatory function of GPR109A has been reported, the specific mechanism of how GPR109A acts as an anti-inflammatory agent and enhances the blood milk barrier is not clear. Until this study, there were no available reports showing that GPR109A plays a role in autophagy, the blood milk and barrier mastitis.

To further explore the biological function of GPR109A, we found that GPR109A activation could significantly enhance the blood milk barrier and alleviate mastitis in WT mice. The level of autophagy and the integrity of the blood milk barrier in GPR109A^-/-^ mice were both significantly inhibited, while the level of inflammation was significantly increased, indicating that the anti-inflammatory function of GPR109A in the mammary gland might be closely related to autophagy. We also found that niacin significantly inhibited mastitis and enhanced the blood milk barrier, but these effects disappeared after GPR109A was knocked out, suggesting that niacin could alleviate inflammation and enhance the blood milk barrier by activating GPR109A. This result supported the hypothesis that GPR109A could reduce inflammation and protect blood milk barrier.

Some studies have shown that both autophagy and Nrf2 can alleviate inflammation 43. Autophagy can alleviate the inflammatory response by removing necrotic or aged organelles and foreign bodies from cells. Autophagy can be divided into noncanonical and canonical pathways 44. In our study, GPR109A was found to significantly promote the phosphorylation of AMPK, so we detected the phosphorylation of Beclin and ULK. The results showed that GPR109A could activate p-Beclin and p-ULK through p-AMPK. We also observed an increase in the flow of autophagy after applying niacin. However, the flow of autophagy significantly decreased after GPR109A was knocked down. Although previous studies have shown that autophagy can alleviate inflammation 45, there is no available study on GPR109A and autophagy.

We found that GPR109A could alleviate mastitis and enhance the blood milk barrier by activating autophagy. Nrf2 is a very important antioxidant related transcription factor. Nrf2 can alleviate inflammation by promoting the expression of its downstream anti-inflammatory genes 46. The Nrf2/HO-1 signaling could significantly reduce various inflammation according to many studies 47. Lv et al. found that isoliquiritigenin can promote the phosphorylation of AMPK and Nrf2 nuclear import in RAW264.7 cells to alleviate lung injury 48. Our study found that GPR109A could also increase the expression of HO-1 through phosphorylation of AMPK in Eph4-Ev. Our experiments showed that GPR109A can exert its anti-inflammatory function through autophagy and Nrf2 and that this function is mainly realized by activating the AMPK signaling pathway.

The blood milk barrier, which plays an important role during lactation, is very important in mother and baby. Some studies have shown that autophagy can enhance the tight junction of the intestinal epithelium 49. Other studies have shown that a lack of Nrf2 can seriously damage the esophagus of mice 50. These studies indicate that Nrf2 also participates in the repair of the barrier. Our research also found that GPR109A can enhance the barrier function through autophagy and Nrf2. Interestingly, we found that the protection of the blood milk barrier by autophagy is better than that mediated by Nrf2. Since niacin promotes autophagy and Nrf2 nuclear import, autophagy and Nrf2 play a synergistic role in enhancing the blood milk barrier.

To further clarify the anti-inflammatory function of GPR109A, we found that different concentrations of niacin could significantly reduce the expression of pro-inflammatory cytokines in RAW264.7 cells. Interestingly, we found that the supernatant of LPS treated RAW264.7 cells could also promote the inflammatory response in mammary epithelial cells, while GPR109A could significantly inhibit the expression of pro-inflammatory cytokines caused by the supernatant (S 3-S 5).

**Figure 11:**
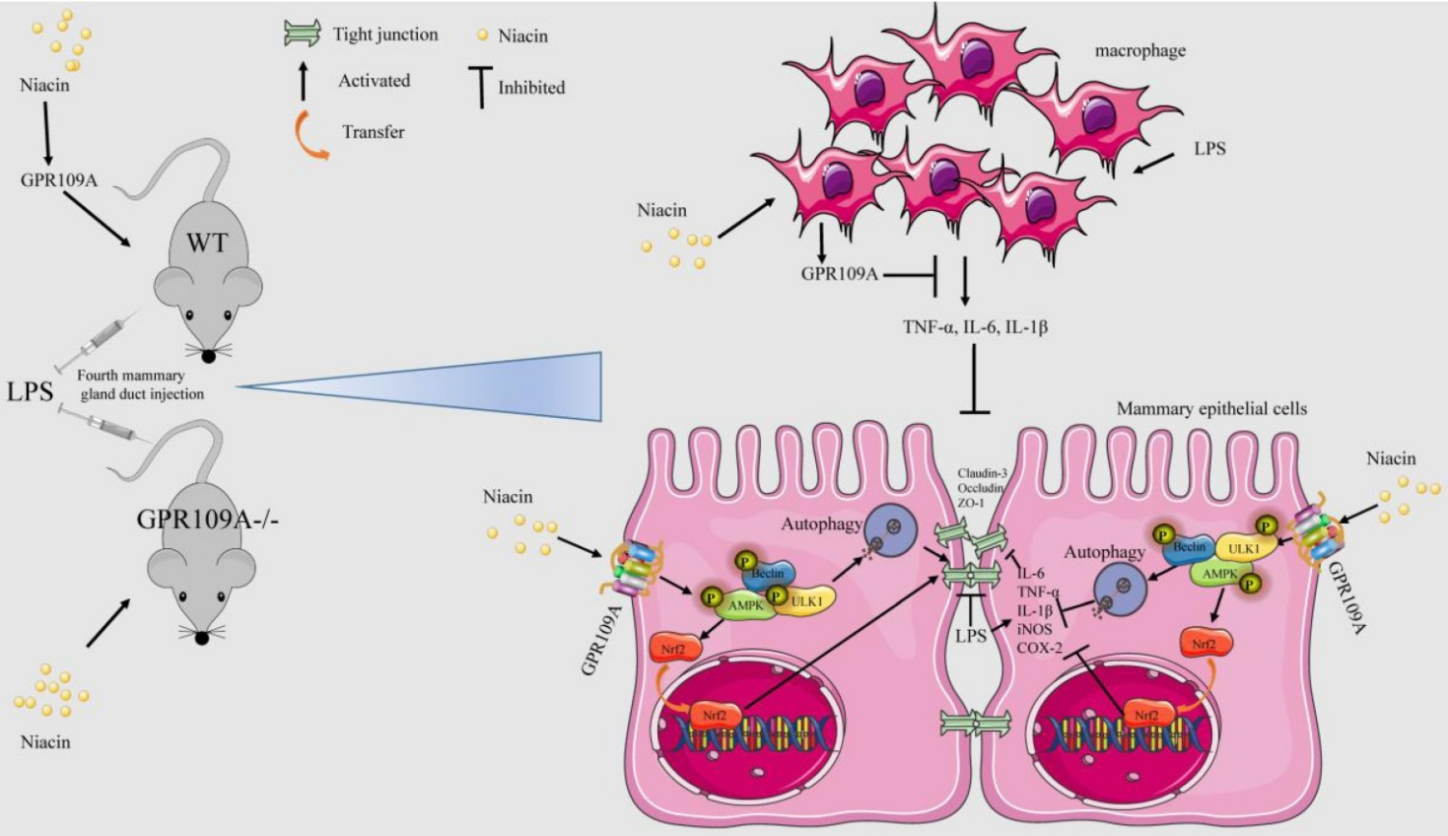
** The mechanism of niacin in anti-mastitis and enhanced blood milk barrier.** GPR109A can alleviate mastitis mainly through two ways. On the one hand, it can alleviate the inflammatory response of mammary epithelial cells and protect the integrity of blood milk barrier by activating AMPK/Nrf2 and autophagy. On the other hand, it can reduce the damage of mammary epithelial cells by inhibiting the release of pro-inflammatory mediators by macrophages.

In conclusion, our results showed that niacin can target GPR109A to alleviate mastitis and enhance the blood milk barrier through AMPK/Nrf2 and AMPK/autophagy. Moreover, GPR109A can significantly reduce the damage to mammary epithelial cells caused by the macrophage supernatant (Fig. 11).

## Supplementary Material

Supplementary figures.

## Figures and Tables

**Table 1:** Primer sequences of* TNF-α, iNOS, IL-6, IL-1β* and *COX-2.*

Item	Primer	Length (bp)	Accession number
*IL-6* (sense)	5'-CCGGAGAGGAGACTTCACAG-3'	134	NM_031168
*IL-6* (anti-sense)	5'- CAGAATTGCCATTGCACAAC-3'		
*TNF-α* (sense)	5'-ACGGCATGGATCTCAAAGAC-3'	116	NM_013693
*TNF-α* (anti-sense)	5'-GTGGGTGAGGAGCACGTAGT-3'		
*IL-1β* (sense)	5'-GCTGCTTCCAAACCTTTGAC-3'	121	NM_008361
*IL-1β* (anti-sense)	5'-AGCTTCTCCACAGCCACAAT-3'		
*COX-2* (sense)	5'-AGAGTCAGTTAGTGGGTAGT-3'	170	NM_011198
*COX-2* (anti-sense)	5'-CTTGTAGTAGGCTTAAACATAG-3'		
*iNOS* (sense)	5'-TCAGACCAAGATCAAGAGCGTGTTG-3'	128	NM_010927
*iNOS* (anti-sense)	5'-GGAACTGTTGAGGAGCGGACAAG-3'		
*β-actin* (sense)	5'-GTCAGGTCATCACTATCGGCAAT-3'	147	NM_007393
*β-actin* (anti-sense)	5'-AGAGGTCTTTACGGATGTCAACGT-3'		

**Table 2:** Antibodies

Item	Company	Country
p-ULK (ser555), ULK, p-Beclin, Beclin, LC3B, AMPK, p-AMPK	Cell Signaling Technology	USA
Claudin 3, HO-1, Nrf2	Abcam	USA
Occludin	Thermo Fisher	USA
Lamin B, ZO-1, P62	Proteintech	USA
β-Tubulin	Bosterbio	USA
IgG	Beyotime	China
Donkey anti-rabbit IgG (H+L)	Life Technologies	USA
HRP-conjugated antimouse and antirabbit secondary antibodies	Bosterbio	USA
