# Supplementary material for: GPR109A alleviate mastitis and enhances the blood milk barrier by activating AMPK/Nrf2 and autophagy
Source: Int J Biol Sci. 2021 Oct 17;17(15):4271–84. doi: 10.7150/ijbs.62380 (PMC8579459; doi:10.7150/ijbs.62380)
Supplement: Supplementary file 1 — Supplementary figures. [file ijbsv17p4271s1.pdf]

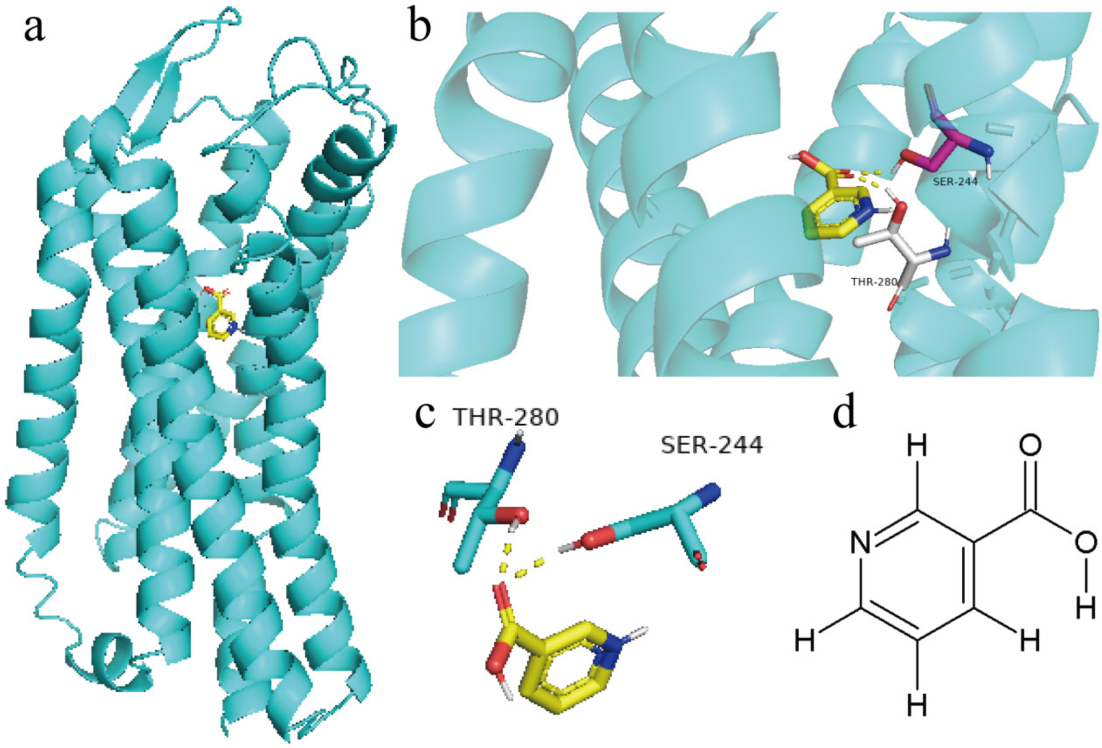

S 1. Active binding sites of niacin with GPR109A

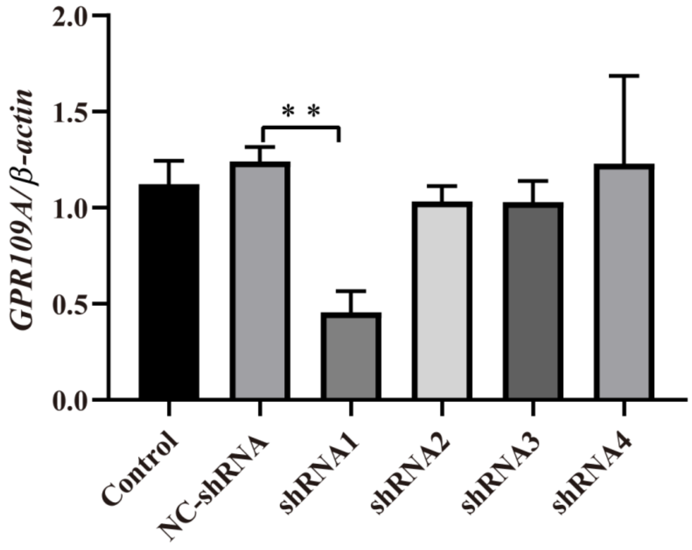

S 2. Knockdown efficiency of GPR109A knockdown plasmid. We designed four GPR109A knockdown sequences (shRNA1, shRNA2, shRNA3 and shRNA4). ShRNA1 has the highest efficiency.

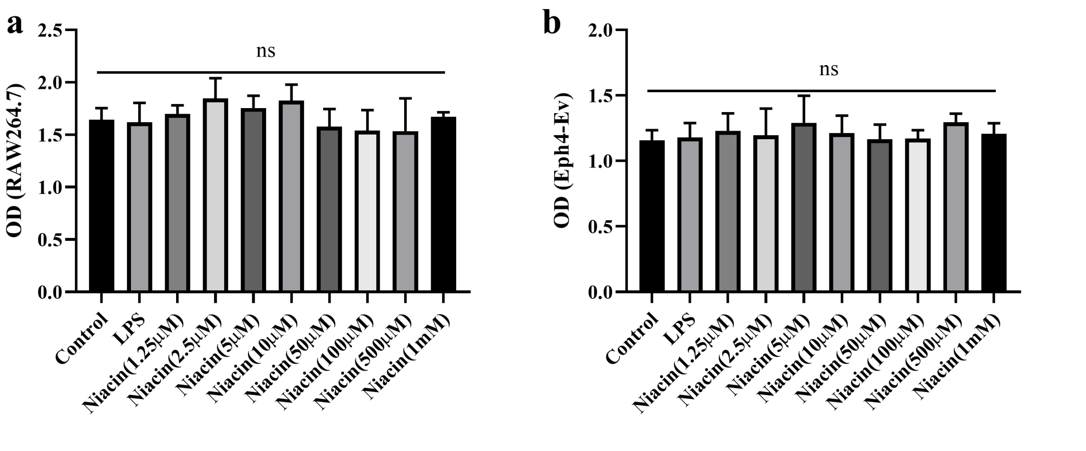

S 3. Cytotoxic experiment of niacin. (a) Toxicity test of niacin in RAW264.7. (b) Toxicity of niacin in EpH4-Ev. Values are presented as means ± SD.

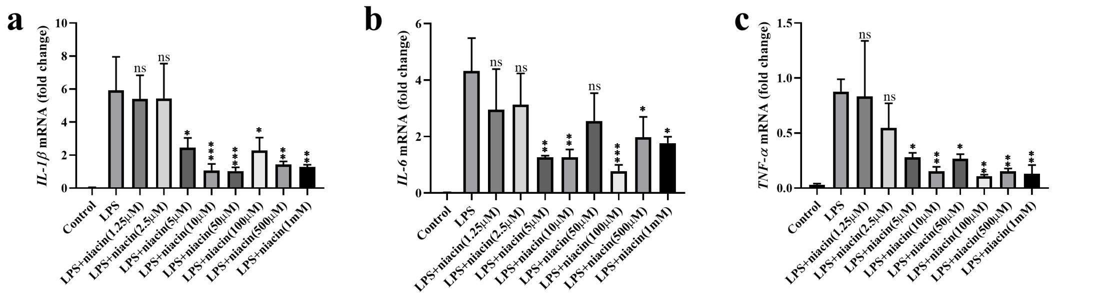

S 4. Niacin reduces the inflammatory response of RAW264.7. (a-c) Gene levels of *IL-6, TNF-α and IL-1β*. Values are presented as means ± SD. (n=3) ($*p < 0.05$ vs LPS, $**p < 0.01$ vs LPS, $***p < 0.001$ vs LPS, $****p < 0.0001$ vs LPS).

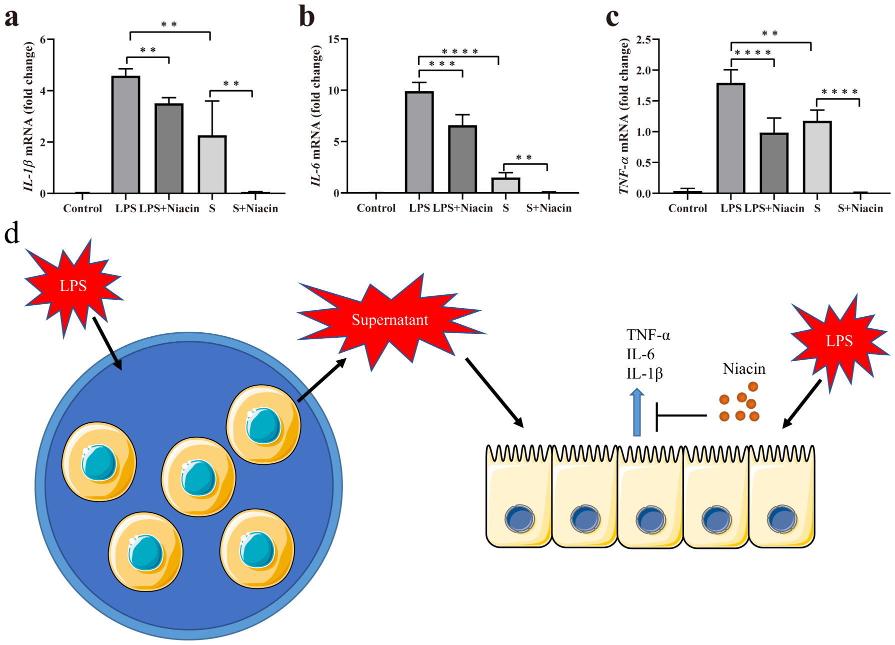

S 5. Niacin can alleviate the inflammation of EpH4-Ev caused by RAW264.7 supernatant. (a-c) Gene levels of *IL-6, TNF-α* and *IL-1β*. Values are presented as means ± SD. (n=3) (**$p < 0.01$, ***$p < 0.001$, ****$p < 0.0001$).
